# Association of *CETP* Gene Polymorphisms and Haplotypes with Acute Heart Rate Response to Exercise

**DOI:** 10.3390/ijms25168587

**Published:** 2024-08-06

**Authors:** Habib Al Ashkar, Nóra Kovács, Ilona Veres-Balajti, Róza Ádány, Péter Pikó

**Affiliations:** 1 Hungarian Research Network University of Debrecen, Public Health Research Group, Department of Public Health and Epidemiology, Faculty of Medicine, University of Debrecen, 4032 Debrecen, Hungary; habib.al.ashkar@med.unideb.hu (H.A.A.); kovacs.nora@med.unideb.hu (N.K.); 2 Department of Public Health and Epidemiology, Faculty of Medicine, University of Debrecen, 4032 Debrecen, Hungary; 3 Department of Physiotherapy, Faculty of Health Sciences, Institute of Health Sciences, University of Debrecen, 4028 Debrecen, Hungary; balajti.ilona@etk.unideb.hu; 4 National Laboratory for Health Security, Center for Epidemiology and Surveillance, Semmelweis University, 1089 Budapest, Hungary; 5 Department of Public Health, Semmelweis University, 1089 Budapest, Hungary

**Keywords:** cardiovascular risk, haplotype, acute heart rate response, cholesteryl ester transfer protein, genomics, single nucleotide polymorphism

## Abstract

Polymorphisms in the cholesteryl ester transfer protein (*CETP*) gene are known to be strongly associated with increased cardiovascular risk, primarily through their effects on the lipid profile and consequently on atherosclerotic risk. The acute heart rate response (AHRR) to physical activity is closely related to individual cardiovascular health. This study aimed to investigate the effect of *CETP* gene polymorphisms on AHRR. Our analysis examines the association of five single nucleotide polymorphisms (SNPs; rs1532624, rs5882, rs708272, rs7499892, and rs9989419) and their haplotypes (H) in the *CETP* gene with AHRR in 607 people from the Hungarian population. Individual AHRR in the present study was assessed using the YMCA 3-min step test and was estimated as the difference between resting and post-exercise heart rate, i.e., delta heart rate (ΔHR). To exclude the direct confounding effect of the *CETP* gene on the lipid profile, adjustments for TG and HDL-C levels, next to conventional risk factors, were applied in the statistical analyses. Among the examined five SNPs, two showed a significant association with lower ΔHR (rs1532624—C_dominant_: B = −8.41, *p* < 0.001; rs708272—G_dominant_: B = −8.33, *p* < 0.001) and reduced the risk of adverse AHRR (rs1532624—C_dominant_: OR = 0.44, *p* = 0.004; rs708272—G_dominant_: OR = 0.43, *p* = 0.003). Among the ten haplotypes, two showed significant association with lower ΔHR (H3—CAGCA: B = −6.81, *p* = 0.003; H9—CGGCG: B = −14.64, *p* = 0.015) and lower risk of adverse AHRR (H3—CAGCA: OR = 0.58, *p* = 0.040; H9—CGGCG: OR = 0.05, *p* = 0.009) compared to the reference haplotype (H1—AGACG). Our study is the first to report a significant association between *CETP* gene polymorphisms and AHRR. It also confirms that the association of the *CETP* gene with cardiovascular risk is mediated by changes in heart rate in response to physical activity, in addition to its effect on lipid profile.

## 1. Introduction

Cholesteryl ester transfer protein (CETP) is a major plasma hydrophobic plasma glycoprotein produced in the liver [1]. It is involved in lipid metabolism and transport by mediating the transfer of triglycerides (TGs) and cholesteryl esters (CEs) in plasma from high-density lipoprotein cholesterol (HDL-C) to apolipoprotein B (apoB)-containing lipoproteins. Plasma CETP levels are positively correlated with atherosclerotic cardiovascular disease (ASCVD) and increase the risk of other types of cardiovascular diseases (CVDs) and premature death [1].

As a result, CETP has been a popular target in pharmaceutical research for the prevention of ASCVD [2]. Studies of CETP inhibitors as drugs to reduce the risk of cardiovascular events have been ongoing for decades [2]. Initially, it was thought that the potential effect of CETP inhibitors was mediated by their ability to increase HDL-C levels, but more recent research suggests that their beneficial effects are primarily due to their ability to reduce the plasma levels of low-density lipoprotein (LDL) cholesterol and apoB [3].

CETP activity has a strong genetic background [4], and certain polymorphisms in the *CETP* gene contribute to the risk of ASCVD [5,6]. Most studies investigating modifications in the *CETP* gene have focused primarily on the effect on lipid profiles and associated diseases [7,8]. In addition, CETP has been associated with mitochondrial oxidant production, respiratory capacity, inflammatory processes, phagocytosis, vascular oxidative stress, and endothelial dysfunction [9,10].

Physical activity has a significant long-term effect on individual lipid profile (dyslipidemia) and, thus, a positive effect on cardiovascular risk [11]. Clinical trials have shown that increased baseline heart rate is associated with cardiovascular morbidity and mortality, independent of other risk factors such as age, gender, diabetes, and hypertension [12,13]. The acute heart rate response (AHRR) to physical activity, which refers to the change in heart rate before and after exercise, is a powerful predictor of major cardiovascular events in both the general population and patients with CVD [14]. Elevated resting heart rate and AHRR are associated with increased progression of atherosclerosis [14] and have been associated with a significantly increased risk of plaque rupture in patients with ASCVD.

The regulation of resting and acute heart rate response to activity is a complex process influenced by autonomic tone, central and peripheral reflexes, and hormonal effects [15]. In addition, it is determined by environmental and lifestyle factors [16] and genetic ones [17,18]. Younger age, male sex, and leisure time physical activity all positively influence AHRR (reduce delta HR) [16,17].

CETP levels are highly genetically determined [19] and are strongly associated with the development of atherosclerosis [20]. The relationship between atherosclerosis and heart rate is well-known and well-studied [12,14,21]. The progression of atherosclerosis negatively affects the flexibility of the blood vessel walls and, in combination, increases heart rate [21]. Nevertheless, no studies have been conducted to investigate the potential association between *CETP* gene variants and AHRR, which are strongly associated with atherosclerosis.

The present study aims to examine the influence of five single nucleotide polymorphisms (SNPs: rs1532624, rs5882, rs708272, rs7499892, and rs9989419) and their haplotypes in the *CETP* gene, which are strongly linked to atherosclerosis progression, on the association with AHRR. In the present study, individual AHRR was assessed using the YMCA 3-min step test [22] and estimated as the difference between the resting heart rate (the heart rate measured one minute before the test after at least two minutes of rest) and the immediate post-exercise heart rate, i.e., the delta heart rate (ΔHR). Additionally, the study aims to either confirm or refute the hypothesis that the potential association is due to the direct effect of *CETP* gene polymorphisms on HDL-C and TG levels.

## 2. Results

### 2.1. Characteristics of the Study Populations, Allele and Haplotype Frequencies by AHRR Groups

After removing records with missing geno- and/or phenotypic data, 607 individuals were included in the present study. For the three groups based on the AHRR (‘adverse’, ‘moderate’, and ‘favorable’), a trend analysis was carried out in comparison to the baseline characteristics. A significant positive trend was observed between the AHRR groups and heart rate immediately after completing the YMCA 3-min step test, delta heart rate (ΔHR), percentage of maximum HR, and optimized polygenic score (for more details, see our previous article [17]), and leisure-time physical activity (in metabolic equivalent minutes per week). There is a significant trend between AHRR groups and the proportion of women, smoking, and education. See Table 1 for more details.

The variation in allele frequencies of the five SNPs and their haplotypes between groups based on AHRR is shown in Supplementary Table S1.

### 2.2. Association of Polymorphisms in the CETP Gene and Their Haplotypes with Heart Rate and Percentage of Maximum Heart Rate

For each polymorphism, we tested which of the three classical inheritance patterns (dominant, co-dominant, and recessive) showed the strongest positive correlation with AHRR. The strongest correlation with HR was shown for rs1532624 with the C allele dominant, rs5882 with the A allele recessive, rs708272 with the G allele dominant, rs7499892 with the C allele recessive and rs9989419 with the G allele recessive inheritance mode. See more details in Supplementary Table S2.

None of the five SNP tested showed after Bonferroni correction a significant correlation with resting heart rate, heart rate 10 min after the physical exercise, and the difference between heart rate 5 and 10 min after exercise. rs1532624 and rs708272 showed a significant association with heart rate measured immediately after physical activity and 5 min later. See Supplementary Table S3 for more details.

Two of the five SNPs tested showed a significant association with AHRR and maximum HR (HR_max_). In a dominant inheritance model, the C allele of rs1532624 showed a significant association with reduced ΔHR (B = −8.41; *p* < 0.001) and percentage of HR_max_ (B = −4.80; *p* < 0.001) and reduced risk of adverse AHRR (OR = 0.44; *p* = 0.004) and reduced risk of adverse targeted heart rate zone—HR_zone_ (OR = 0.35; *p* < 0.001). Similarly, the G allele of rs708272 in a dominant heritability model showed a significant association with reduced ΔHR (B = −8.33; *p* < 0.001) and percentage of HR_max_ (B = −4.67; *p* < 0.001), as well as reduced odds of adverse AHRR (OR = 0.45; *p* = 0.003) and reduced risk of adverse HR_zone_ (OR = 0.34; *p* < 0.001). See Table 2 for more details.

Of the ten haplotypes tested, three (H3, H4, and H9) showed significant associations with at least one of the HR-related parameters. Haplotype H3 showed a significant inverse association with each of the four variables. Haplotype H4 showed a significant association with reduced HR_max_ percentage (B = −2.79, 95%CI: −5.52–−0.05, *p* = 0.046) and the lower risk of adverse HR_zone_ (OR = 0.48, 95%CI: 0.24–0.98, p = 0.046). Haplotype H9 showed a significant association with reduced ΔHR (B = −14.64, 95%CI: −26.39–−2.88, *p* = 0.015), lower risk of adverse AHRR (OR = 0.05, 95%CI: 0.01–0.47, *p* = 0.009), and reduced HR_max_ percentage (B = −7.19, 95%CI: −13.23–−1.15, *p* = 0.020). See Table 3 for more details.

## 3. Discussion

The present study aimed to confirm or refute our hypothesis that *CETP* gene polymorphisms involved in atherosclerotic processes are associated with an alteration of the acute heart rate response to physical activity. In addition, we investigated whether this association, if it exists, is related to the direct effect of *CETP* gene polymorphisms and haplotypes on the lipid profile or whether it is based on independent mechanisms.

Among the five SNPs examined in the current study, rs1532624 and rs708272 showed significant association with AHRR-related parameters. These SNPs show a high degree of linkage, and therefore, their significant association with AHRR is not independent of each other. Based on literature data, both SNPs have been associated with CETP activity [23], HDL-C levels [24,25], Apoprotein A-I concentration [26], and risk of cardiovascular diseases [27,28]

Of the 10 haplotypes tested, three (H3, H4, and H9) showed a significant association with at least one of the AHRR-related parameters. In our previous study [29], H3 was significantly associated with lower HDL-C levels (B = −0.05, 95%CI: −0.09–−0.01, *p* = 0.016), increased risk of reduced HDL-C levels (OR = 1.34, 95%CI: 1.01–1.76, *p* = 0.040), and lower TG levels (B = −0.16, 95%CI. −0.30–−0.01, *p* = 0.033) but not with TG/HDL-C ratios. H4 and H9 did not show a significant association with any of the lipid parameters (HDL-C, TG and TG/HDL-C ratio) investigated in our previous article [29]. Furthermore, none of the identified haplotypes showed a significant association with the estimated cardiovascular risk [5].

To date, no studies have linked polymorphisms in the *CETP* gene and their haplotypes with the acute heart rate response to physical activity. The original hypothesis was that the effect of polymorphisms in the *CETP* gene on AHRR is mediated by CETP protein activity on lipid profile (mainly by altering HDL-C and TG levels) and thus on atherosclerosis. The results of the present study challenge this hypothesis. After controlling for HDL-C and TG levels, the association of *CETP* gene polymorphisms with AHRR remained significant. The results suggest that CETP influences individual AHRRs through a mechanism that is independent of its effect on HDL-C and TG levels.

This study had limitations. Although the YMCA 3-min step test is suitable and widely used to assess changes in heart rate in response to exercise in population-based surveys [30,31,32], the 60-s pulse measurement time proposed in the test protocol and also used in our study to stratify the sample may be too long to estimate post-exercise heart rate, because it declines rapidly. However, it should be noted that our results obtained by testing the association between genomic factors and AHRR are not significantly affected by a possible systemic underestimation of the post-exercise heart rate. Analyses were corrected for relevant covariates; however, several environmental lifestyle and individual factors (thyroid activity and hemoglobin levels) not assessed in the present study may influence heart rate changes induced by physical activity.

Furthermore, some additional factors (epigenetic, rare, or structural variants, gene–gene and/or gene–environment interactions) may also influence and modify the results. A major limitation of the present study is the sample size, which may limit the statistical power. Although the results are still statistically significant after the Bonferroni correction, it would be useful to perform further analyses in a larger sample of different ethnicities to confirm our findings. Furthermore, due to the cross-sectional nature of the present study, the results obtained can be considered as a single time point and therefore, no causal relationship can be concluded between *CETP* gene polymorphisms and acute heart rate changes in response to physical activity, but the results obtained can be considered in creating hypotheses on the relationship between CETP and heart functions.

In conclusion, the present study is the first to describe the effect of *CETP* gene polymorphisms and haplotypes on heart rate changes in response to physical activity. In this study, we have successfully demonstrated an association between polymorphisms in the *CETP* gene and AHRR, independent of lipid profile (TG and HDL-C levels), which may be mediated by other mechanisms related to gene activity (e.g., inflammation, blood pressure, coagulation). It is worth mentioning that in an animal model, *CETP* polymorphism was shown to affect not only serum and adipose tissue lipid metabolism but also muscle lipid metabolism [33]. The involvement of CETP in intramuscular fat deposition was shown in another animal study [34], and it was also demonstrated that in transgenic female mice, CETP can protect against obesity-induced impairment in exercise capacity and may be a target to improve exercise capacity in the context of obesity [35]. In human studies, a reverse correlation has been found between *CETP* gene polymorphisms and/or CETP activity and blood pressure [36,37], endothelial dysfunction [38], large and small vessel strokes [39], and intracerebral hemorrhage [40]. Nevertheless, it is reasonable to suppose that further studies are needed to understand the physiological role of CETP beyond its role in lipid metabolism.

## 4. Materials and Methods

### 4.1. Study Design and Populations

The research design and data collection have been described in detail in a previous paper [41]. Briefly, in 2018, a three-pillar (i.e., questionnaire-based, physical examination, and laboratory examination) complex (i.e., health behavior and examination) cross-sectional survey of the Hungarian general and Roma population aged 20–64 years was conducted in two counties in northeastern Hungary. The questionnaire used in the survey was based on the European Health Interview Survey (EHIS) Wave 2, which consists of four modules (health status, health care utilization, health determinants and socioeconomic measures), and it was extended with some additional sets of questions, including the long version of the International Physical Activity Questionnaire (IPAQ) to measure physical activity by domains and dimensions. A total of 832 randomly selected participants were recruited. Participants’ ethnic background was self-reported.

Fasting blood samples were taken for routine laboratory tests (e.g., total cholesterol, TG, LDL cholesterol, HDL-C, and blood glucose levels) in the framework of the physical examination pillar of the survey, then the anthropometric (e.g., height and weight), demographic (e.g., age and sex), socioeconomic, and health-related data (on medication use, by YMCA 3-min step test, and blood pressure measurement) were also collected. Individuals with incomplete genotype and/or phenotype data were excluded from further analysis.

### 4.2. DNA Isolation, Selection of SNPs, Genotyping Progress, Result of Hardy-Weinberg (HW) and Linkage Disequilibrium (LD) Analyses

As described in our previous study [25], DNA was isolated using the MagNA Pure LC system (Roche Diagnostics, Basel, Switzerland) and MagNA Pure LC DNA Isolation Kit—Large. Genotyping was performed by the Mutation Analysis Core Centre (MAF) at Karolinska University Hospital, Sweden. Genotyping was performed on a MassARRAY platform (Sequenom Inc., San Diego, CA, USA) using iPLEX gold chemistry.

The selection of SNPs under study was based on a literature review (PubMed, HuGE Navigator and Ensembl databases). Aiming to identify SNPs in the *CETP* gene, SNPs whose effects on HDL-C levels were observed in several populations (even of different origin) were included.

There were no significant differences from LD for the five SNPs tested. Based on the HWE analysis [25], there is a linkage between rs1532624 and rs708272.

### 4.3. Measurement of Physical Activity and Acute Heart Rate Response

Physical activity levels were assessed using the International Physical Activity Questionnaire (IPAQ) [42]. The IPAQ assesses the time spent in light, moderate, and vigorous activities in the past week in several domains (work, transport, leisure time, domestic, and gardening). Based on the results of the questionnaire, MET-min/week was calculated for each person.

The YMCA 3-min step test was used to assess individual heart rate changes in response to physical activity. The test starts with a 2-min rest period while the subjects sit on a chair in a quiet room. Subjects are asked to step up and down a 30 cm step/bench 72 times in 3 min without interruption. The cadence of the steps is indicated by a metronome set to 96 beats per minute (4 clicks = one step cycle), which is 24 steps/minute (72 steps/3 min). At the end of the test, subjects shall immediately stop and then sit down and remain motionless. Heart rate was measured four times during the YMCA 3-min step test: one minute before the test at rest (HR_rest_), immediately after the test (HR_excerc_), 5 min (HR_5min_) and 10 min (HR_10min_) after the test. The heart rate was estimated by a health professional by manual pulse palpation in the wrist’s radial artery for 60 s.

The difference between the resting heart rate and the heart rate after the step test was defined as ΔHR. The ΔHR is inversely related to the risk of CVDs, i.e., a lower value indicates better cardiovascular health [43]. Individuals participating in the study were ranked according to ΔHR (from higher to lower) and classified into AHRR groups of adverse, moderate, and favorable. In this study, the Jonckheere–Terpstra trend test [44] was used to compare AHRR groups and identify differences between relevant factors.

Age-related maximum heart rate was calculated to further assess the heart rate response to physical activity. The following formula was used to estimate the maximum heart rate: 220 − age (years) [45]. HR_max_ expressed as a percentage (HR_max%_ = (HR_exerc_/(220 − age in years)) × 100) were calculated to determine and compare target heart rate zones (HR_zone_) [46]. The reference group included subjects whose HR_max%_ was less than or equal to 64% (*n* = 379), while the adverse group (*n* = 104) included subjects whose HR_max%_ was greater than 76%.

### 4.4. Statistical Analysis

IBM SPSS (version 27, IBM Company, Armonk, NY, USA) and SNPStats online tools (https://www.snpstats.net/start.htm, accessed on 24 April 2024) [47] were used for statistical analyses. The Shapiro–Wilk test was employed to assess the normality of the distribution of quantitative variables, and Templeton’s two-step method was used to normalize variables when applicable [48]. χ^2^ test was used to compare non-quantitative variables.

To examine the individual effects of SNPs, the three most used heritability models (dominant, recessive, and co-dominant) were evaluated using the *p*-value, the Akaike Information Criterion (AIC) and the Bayesian Information Criterion (BIC). The heritability model, for which the statistical model was most optimal, was selected for further analysis. To study the association between the SNPs and their haplotypes, with the AHRR-related parameters multivariate linear and logistic regression analyses were performed. All statistical analyses were adjusted for ethnicity, sex, age, traveling by vehicle, leisure-time physical activity in metabolic equivalent task minutes per week (MET-min/week), body mass index (BMI), education, diastolic blood pressure, fasting glucose, current smoking status, HDL-C, TG levels, antihypertensive, antidiabetic, and lipid-lowering medication, history of heart-related disease and optimized polygenic score for AHRR [17].

In all analyses, except for the individual effect of SNPs, *p* < 0.05 was considered significant. For the analysis of the individual effect of SNPs (due to the high LD value of rs1532624 and rs708272), a Bonferroni-corrected *p*-value (conventional *p*-value divided by the number of independent SNPs: *p* = 0.05/4 = 0.0125) was considered significant.

## Figures and Tables

**Table 1:** Basic characteristics of the three groups based on acute heart rate response (AHRR—assessed as the difference between post-exercise and resting heart rate—delta heart rate—ΔHR).

		Adverse AHRR (*n* = 200)	Moderate AHRR (*n* = 203)	Favorable AHRR (*n* = 204)	*p* for Trend
		Mean (95%CI)			
HR_rest_		76.69 (75.38–78.01)	76.81 (75.40–78.22)	78.50 (77.05–79.95)	0.152
HR_exerc_		138.78 (134.42–141.13)	103.37 (101.86–104.88)	91.15 (89.55–92.76)	<0.001 *
ΔHR ^¥^		61.08 (57.84–64.32)	26.56 (25.99–27.17)	12.73 (12.07–13.38)	<0.001 *
Percentage of maximum heart rate (%)		77.86 (75.92–79.81)	58.90 (57.93–59.86)	51.67 (50.59–52.76)	<0.001 *
oPGS		6.68 (6.33–7.03)	7.71 (7.42–8.00)	7.65 (7.33–7.97)	<0.001 *
Age (years)		42.39 (40.60–44.18)	43.83 (42.16–45.50)	42.60 (40.88–44.32)	0.813
Body mass index (kg/m^2^)		27.50 (26.59–28.40)	27.33 (26.58–28.08)	26.86 (26.05–27.67)	0.355
Average diastolic blood pressure (mmHg)		79.83 (78.61–81.06)	78.64 (77.45–79.83)	79.13 (77.72–80.54)	0.504
Fasting glucose (mmol/L)		5.04 (4.84–5.23)	5.08 (4.86–5.30)	5.10 (4.89–5.30)	0.910
TG (mmol/L)		1.54 (1.39–1.68)	1.51 (1.38–1.63)	1.62 (1.47–1.76)	0.459
HDL-C (mmol/L)		1.32 (1.27–1.37)	1.33 (1.28–1.39)	1.29 (1.24–1.34)	0.569
Leisure-time physical activity (MET-min/week)		830.07 (625.01–1035.13)	1349.13 (1068.99–1630.36)	1440.13 (1177.58–1702.68)	<0.001 *
		Prevalence in % (95%CI)			*p* for trend
Women		72.50 (66.02–78.33)	64.04 (57.28–70.40)	60.29 (53.47–66.82)	0.010 *
Traveling by vehicle		45.00 (38.22–51.93)	59.11 (52.26–65.71)	47.06 (40.26–53.91)	0.698
Education	Primary	55.00 (48.07–61.78)	38.42 (31.93–45.24)	63.73 (56.97–70.09)	0.026 *
	High school	31.50 (25.36–38.17)	51.27 (44.87–58.53)	29.41 (23.48–35.92)	
	University	13.50 (9.30–18.75)	9.85 (6.32–14.52)	6.89 (3.99–10.95)	
Current smoking status		48.00 (41.15–54.91)	40.39 (33.82–47.24)	57.84 (51.00–64.47)	0.045 *
Roma ethnicity		53.50 (46.58–60.32)	31.53 (25.43–38.15)	61.76 (54.97–68.23)	0.087

^¥^: The parameter used to create the subgroups; 95%CI—95% confidence interval; *: *p* < 0.05; oPGS—optimized polygenic score [17]; HR_rest_—resting heart rate; HR_exerc_—heart rate immediately after completing the YMCA 3-min step test.

**Table 2:** Association of SNPs in the *CETP* gene with acute heart rate response (AHRR—assessed as the difference between post-exercise and resting heart rate—delta heart rate—ΔHR) as continuous and binary (favorable AHRR was used as reference) outcome variable; and with the percentage of maximum heart rate (HR_max_) as continuous and binary and binary (targeted heart rate zone—HR_zone_: HR_max_: ≤64%—as reference) vs. HR_max_: >76%) outcome variable.

	ΔHR		Risk of Adverse AHRR	
	B (95%CI)	*p*-Value	OR (95%CI)	*p*-Value
rs1532624 (C—dominant)	−8.41 (−13.10–−3.72)	<0.001 **	0.44 (0.26–0.78)	0.004 **
rs5882 (A—recessive)	−4.61 (−8.58–−0.64)	0.023 *	0.77 (0.49–1.20)	0.250
rs708272 (G—dominant)	−8.33 (−12.84–−3.83)	<0.001 **	0.45 (0.26–0.77)	0.003 **
rs7499892 (C—recessive)	1.34 (−2.72–5.39)	0.520	1.04 (0.66–1.64)	0.850
rs9989419 (G—recessive)	2.36 (−1.57–6.29)	0.240	1.32 (0.85–2.04)	0.220
	**Percentage HR_max_**		**Risk of Adverse HR_zone_**	
	**B (95%CI)**	***p*-Value**	**OR (95%CI)**	***p*-Value**
rs1532624 (C—dominant)	−4.80 (−7.44–−2.17)	<0.001 **	0.35 (0.19–0.64)	<0.001 **
rs5882 (A—recessive)	−1.90 (−4.14–0.33)	0.096	0.56 (0.32–0.98)	0.039 *
rs708272 (G—dominant)	−4.67 (−7.20–−2.14)	<0.001 **	0.34 (0.19–0.61)	<0.001 **
rs7499892 (C—recessive)	0.76 (−1.52–3.03)	0.520	1.16 (0.66–2.04)	0.610
rs9989419 (G—recessive)	1.20 (−1.01–−3.41)	0.290	1.43 (0.84–2.44)	0.180

All analyses were adjusted for ethnicity, sex, age, traveling by vehicle, leisure-time physical activity in metabolic equivalent task minutes per week (MET-min/week), body mass index (BMI), education, diastolic blood pressure, fasting glucose, current smoking status, HDL-C, TG, antihypertensive, antidiabetic and lipid-lowering medication, history of heart-related disease and optimized polygenic score for AHRR [17]. The effect allele and heritability model of SNPs are shown in brackets. 95%CI—95% confidential interval, *: *p* < 0.05, **: *p* < 0.0125 (Bonferroni-corrected).

**Table 3:** Association of haplotypes (H) in the *CETP* gene with acute heart rate response (AHRR—assessed as the difference between post-exercise and resting heart rate—delta heart rate—ΔHR) as continuous and binary (favorable AHRR was used as reference) outcome variable; and with percentage of maximum heart rate (HR_max_) as continuous and binary (targeted heart rate zone—HR_zone_: HR_max_: ≤64%—as reference) vs. HR_max_: >76%) outcome variable—B.

	H1	H2	H3	H4	H5	H6	H7	H8	H9	H10
rs1532624	A	A	C	C	C	C	A	C	C	C
rs5882	G	A	A	A	A	G	A	G	G	A
rs708272	A	A	G	G	G	G	A	G	G	G
rs7499892	C	C	C	C	T	C	C	T	C	T
rs9989419	G	G	A	G	A	A	A	G	G	G
ΔHR	Ref.	N.S.	B = −6.81 95%CI: −11.38–−2.25 *p* = 0.003	N.S.	N.S.	N.S.	N.S.	N.S.	B = −14.64 95%CI: −26.39–−2.88 *p* = 0.015	N.S.
Risk of adverse AHRR	Ref.	N.S.	OR = 0.58 95%CI: 0.34–0.97 *p* = 0.040	N.S.	N.S.	N.S.	N.S.	N.S.	OR = 0.05 95%CI: 0.01–0.47 *p* = 0.009	N.S.
Percentage HR_max_	Ref.	N.S.	B = −3.35 95%CI: −5.97–−0.73 *p* = 0.012	B = −2.79 95%CI: −5.52–−0.05 *p* = 0.046	N.S.	N.S.	N.S.	N.S.	B = −7.19 95%CI: −13.23–−1.15 *p* = 0.020	N.S.
Risk of adverse HR_zone_	Ref.	N.S.	OR = 0.27 95%CI: 0.12–0.58 *p* = 0.001	OR = 0.48 95%CI: 0.24–0.98 *p* = 0.046	N.S.	N.S.	N.S.	N.S.	Low sample size ^€^	N.S.

All analyses were adjusted for ethnicity, sex, age, traveling by vehicle, leisure-time physical activity in metabolic equivalent task minutes per week (MET-min/week), body mass index (BMI), education, diastolic blood pressure, fasting glucose, current smoking status, HDL-C, TG, antihypertensive, antidiabetic and lipid-lowering medication, history of heart-related disease and optimized polygenic score for AHRR [17]. Ref.: reference haplotype; N.S.: not statistically significant; ^€^: statistical calculation is not possible due to the small sample size.

## Data Availability

Data are available on request due to privacy or ethical concerns.

## Supplementary Materials

The following supporting information can be downloaded at: https://www.mdpi.com/article/10.3390/ijms25168587/s1.
