# Supplementary material for: Association of CETP Gene Polymorphisms and Haplotypes with Acute Heart Rate Response to Exercise
Source: Int J Mol Sci. 2024 Aug 6;25(16):8587. doi: 10.3390/ijms25168587 (PMC11354538; doi:10.3390/ijms25168587)
Supplement: Supplementary file 1 [file ijms-25-08587-s001.zip › Supplementary Table S1.pdf]

Supplementary Table S1. The distribution of minor and major allele frequencies of the five SNPs and their haplotypes by acute heart rate response (AHRR—assessed as the difference between post-exercise and resting heart rate—delta heart rate—ΔHR) categories.

	Total population (n = 607)	Adverse AHRR (n = 200)	Moderate AHRR (n = 203)	Favorable AHRR (n = 204)
SNPs (minor/major allele)		Prevalence in %		
rs1532624 (A/C)	44/56	46/54	45/55	41/59
rs5882 (G/A)	37/63	40/60	35/65	37/63
rs708272 (A/G)	45/55	47/53	46/54	42/58
rs7499892 (T/C)	21/79	20/80	20/80	22/78
rs9989419 (G/A)	63/37	64/36	63/37	61/39
Haplotypes (H)		Prevalence in %		
H1 (AGACG)	23.82	28.63	21.89	21.10
H2 (AAACG)	16.41	14.63	17.85	16.53
H3 (CAGCA)	13.41	11.72	13.14	15.08
H4 (CAGCG)	12.06	12.84	12.42	11.15
H5 (CAGTA)	13.26	12.91	12.99	13.75
H6 (CGGCA)	5.67	7.50	3.27	6.35
H7 (AAACA)	2.63	2.48	3.53	3.04
H8 (CGGTG)	3.18	2.40	3.00	4.62
H9 (CGGCG)	2.62	0.44	3.40	3.93
H10 (CAGTG)	3.42	3.90	2.79	2.94
