# Supplementary material for: Association of CETP Gene Polymorphisms and Haplotypes with Acute Heart Rate Response to Exercise
Source: Int J Mol Sci. 2024 Aug 6;25(16):8587. doi: 10.3390/ijms25168587 (PMC11354538; doi:10.3390/ijms25168587)
Supplement: Supplementary file 1 [file ijms-25-08587-s001.zip › Supplementary Table S2.pdf]

Supplementary Table S2. Statistical model accuracy assessment based on *p*-value, Akaike information criterion (AIC), and Bayesian information criterion (BIC) for the association of the five SNPs with AHRR according to the classical three inheritance modes (codominant, recessive, and dominant).

SNP (effect allele)	Codominant	Recessive	Dominant
		p-value (AIC/BIC)	
rs1532624 (C)	0.003 (5569/5648)	0.340 (5578/5653)	<0.001 (5567/5642) ∞
rs5882 (A)	0.063 (5575/5654)	0.022 (5573/5648) ∞	0.740 (5579/5654)
rs708272 (G)	0.001 (5567/5647)	0.510 (5578/5653)	<0.001 (5566/5641) ∞
rs7499892 (C)	0.820 (5580/5660)	0.540 (5578/5653) ∞	0.910 (5579/5654)
rs9989419 (G)	0.500 (5579/5659)	0.240 (5577/5652) ∞	0.800 (5579/5654)

∞: most optimal inheritance/statistical model
