# Supplementary material for: Association of CETP Gene Polymorphisms and Haplotypes with Acute Heart Rate Response to Exercise
Source: Int J Mol Sci. 2024 Aug 6;25(16):8587. doi: 10.3390/ijms25168587 (PMC11354538; doi:10.3390/ijms25168587)
Supplement: Supplementary file 1 [file ijms-25-08587-s001.zip › Supplementary Table S3.pdf]

Supplementary Table S3. Association of the five SNPs in the *CETP* gene with heart rate

	HR_{rest}	HR_{exerc}	HR_{5min}	HR_{10min}	ΔHR_{5min}	ΔHR_{10min}
	B (95%CI)					
rs1532624 (C - dominant)	-0.51 (-2.18 – 1.16)<br>*p* =0.550	-7.81 (-12.53 – -3.08)<br>*p* = 0.001**	-4.17 (-7.16 – -1.19)<br>*p* = 0.006**	-2.56 (-4.70 – -0.41)<br>*p* = 0.020*	-3.29 (-6.18 – -0.39)<br>*p* = 0.026*	-1.44 (-3.16 – 0.27)<br>*p* = 0.100
rs5882 (A - recessive)	0.81 (-0.78 – 2.39)<br>*p* = 0.320	-3.57 (-7.50 – 0.36)<br>*p* = 0.076	-1.29 (-3.78 – 1.189)<br>*p* = 0.310	0.42 (-1.36 – 2.20)<br>*p* = 0.650	-2.16 (-4.56 – 0.23)<br>*p* = 0.078	-0.18 (-1.61 – 1.25)<br>*p* = 0.810
rs708272 (G - dominant)	-1.00 (-2.83 – 0.84)<br>*p* = 0.290	-7.65 (-12.17 – -3.12)<br>*p* = 0.001**	-4.18 (-7.04 – -1.32)<br>*p* = 0.004**	-2.50 (-4.55 – -0.45)<br>*p* = 0.017*	-3.33 (-6.10 – -0.56)<br>*p* = 0.019*	-1.41 (-3.06 – 0.24)<br>*p* = 0.094
rs7499892 (C - recessive)	0.37 (-1.27 – 2.01)<br>*p* = 0.660	1.05 (-3.02 – 5.13)<br>*p* = 0.610	1.30 (-1.28 – 3.87)<br>*p* = 0.320	0.16 (-1.68 – 2.00)<br>*p* = 0.870	1.34 (-1.14 – 3.83)<br>*p* = 0.290	0.45 (-1.02 – 1.93)<br>*p* = 0.550
rs9989419 (G - recessive)	-0.86 (-3.04 – 1.32)<br>*p* = 0.440	2.10 (-1.86 – 6.05)<br>*p* = 0.300	1.40 (-1.09 – 3.90)<br>*p* = 0.270	-0.07 (-1.85 – 1.72)<br>*p* = 0.940	1.50 (-0.91 – 3.91)<br>*p* = 0.220	-0.09 (-1.52 – 1.34)<br>*p* = 0.910

HR_{rest}: resting heart rate; HR_{exerc}: heart rate immediately after completing the physical exercise; HR_{5min}: heart rate 5 minutes after the physical exercise; HR_{10min}: heart rate 10 minutes after the physical exercise; ΔHR: delta heart rate defined as the difference between the heart rate immediately after completing the physical exercise and the resting heart rate; ΔHR_{5min}: defined as the difference between the heart rate 5 minutes after physical exercise and the resting heart rate; ΔHR_{10min}: defined as the difference between the heart rate 10 minutes after physical exercise and the resting heart rate. 95%CI: 95% confidence interval; *: p <0.05, **: p <0.0125 (Bonferroni corrected).
